# Microstructure and Mechanical Properties of Al/Steel Butt Joint by Hybrid CMT Welding with External Axial Magnetic Field

**DOI:** 10.3390/ma13163601

**Published:** 2020-08-14

**Authors:** Kexin Kang, Yibo Liu, Junzhao Li, Chao Liu, Zuyang Zhen, Yaxin Wang, Qingjie Sun

**Affiliations:** 1 Shandong Provincial Key Laboratory of Special Welding Technology, Harbin Institute of Technology at Weihai, No.2 West Wenhua Road, Weihai 264209, China; kangkx_hit@163.com (K.K.); ljzhao_hit@163.com (J.L.); 17863109157@163.com (C.L.); zhenzuyang@163.com (Z.Z.); wangyaxin1995@163.com (Y.W.); qjsun@hit.edu.cn (Q.S.); 2 State Key Laboratory of Advanced Welding and Joining, Harbin Institute of Technology, No.92 West Dazhi Street, Harbin 150001, China

**Keywords:** axial magnetic field, aluminum and steel, CMT welding, interfacial microstructure, tensile strength

## Abstract

The 6061 aluminum alloy and 304 stainless steel were welded by hybrid cold metal transfer (CMT) welding with external axial magnetic field. The effects of magnetic intensity and frequency on joint microstructure and mechanical properties were studied. It was found that the magnetic field can promote the spreading of aluminum weld metal on the steel surface and thus increase the bonding area of Al/steel butt joint. The welding process stability improved, while the wetting behavior worsened with the introduction of alternating frequencies. The thickness of the intermetallic compound (IMC) layer at Al/steel interface was reduced to 3 μm with the coil current of 2 A. The application of the magnetic field promoted the aggregation of Si atoms at the interface and inhibited the formation of brittle (Al, Si)_13_Fe_4_ phase. The fracture paths were transformed from (Al, Si)_13_Fe_4_ layer to Al_8_Fe_2_Si layer with the application of the magnetic field. The maximum tensile strength reached 130.2 MPa, an increase of 61.6% in comparison to the normal CMT process.

## 1. Introduction

In order to save fossil energy and reduce greenhouse gas emissions, the lightweight automobile has become one of the development directions of automobile industry. Due to its high specific strength, good corrosion resistance, and low cost, aluminum and its alloys have great potential to replace the conventional steel component. As a result, the sound aluminum and steel welded joint has gained extensive attention [1]. However, distinct differences in thermal physical properties and the formations of brittle intermetallic compounds (IMCs) at the bonding interface are obstacles to achieve effective connection [2]. Therefore, many welding methods have been applied to realize dissimilar metals welding of Al and steel [3]. The key of Al/steel welding is to control the IMC layer thickness at the interface. Chen et al. [4] considered that only when the thickness of IMC layer is less than 10 μm will the joints achieve good properties. Cao et al. [5] analyzed the relationship between joint strength and IMCs and found that the tensile strength of the joint decreased when the IMC layer was thicker than 5 μm.

At present, two main methods are used to control the growth of the IMC layer. One is to add alloy elements at the interface. Zn addition is known to change the IMC phase, which can decrease the brittleness of the interfacial region and consequently increase the fracture load [6,7,8]. He et al. [9] found that the addition of Ni to 5A06 aluminum alloy and SUS321 stainless steel welding can significantly decrease the IMC layer thickness and improve the mechanical properties of the butt joints. Si is considered to be able to decelerate growth and reduce the brittleness of the IMC layer, thereby improving the joint mechanical properties [10,11,12,13]. Xia et al. [14] joined 6061-T6 aluminum alloy and DP590 steel by the laser welding-brazing process with pure Al, AlSi5, and AlSi12 filler metals. They found that Si content affected the reaction mechanism and led to different IMC components. The joint produced with the AlSi5 filler metal had the highest tensile strength and the largest fracture displacement. Moreover, it is important to control the heat input of Al/steel welding process. It is acknowledged that lower heat input is beneficial to the inhibition of IMC growth and the improvement of joint strength [15,16]. Shiran et al. [17] investigated the effect of heat treatment on the IMCs of the stainless steel 321-aluminum 1230 explosive-welding interface. The results indicated that the IMC layer thickness increased and the joint strength decreased with the increase of the duration and temperature of heat treatment. Li et al. [18] found that with the increase of heat input, the thickness and the brittleness of the IMC layer increased accordingly. However, low heat input tended to result in terrible wettability of welding seam due to the bad fluidity of the molten pool with a low temperature [19,20].

Nevertheless, the wetting behavior was considered to have a strong influence on the mechanical strength of the assembly [21]. Sun et al. [22] joined Al/steel with ER4043 filler metal and found that the tensile strength of the Al/steel butt joint is not only related to the thickness of the IMC layer, but also the bonding area. Therefore, improving the wettability of the joint and reducing the thickness of the IMC layer are two avenues to obtain the high tensile strength of Al/steel butt joint. As an auxiliary means of arc welding, the magnetic field has played an important role in the welding process with no solder assisted [23]. Yan et al. [24] discovered that the magnetic field can control the formation of IMCs at the interface of Al/steel and inhibit the crack growth in the laser welding process. Sun et al. [25] joined aluminum and titanium by cold metal transfer (CMT) welding process and found that the heating area of weld arc increased and the peak temperature reduced with the adaptation of axial magnetic field. Jin et al. [26] found that the magnetic field can promote the wetting of liquid Cu on a steel surface and affect the interfacial microstructure evolution in CMT welding process.

Therefore, in the present research, as a welding method characterized by low heat input [27], CMT welding with an external axial magnetic field was adopted to join 6061-T6 aluminum alloy and 304 stainless steel. The purpose of this study is to reveal the effect of the external axial magnetic field on wetting behavior, interfacial microstructure, and mechanical properties.

## 2. Materials and Methods 

In this study, 6061-T6 aluminum alloy and 304 stainless steel plates with dimensions of 100 mm × 100 mm × 2 mm were chosen as base metals. The tensile strength of SS304 and 6061-T6 substrates were 716 MPa and 262 MPa, respectively. The filler metal adapted was ER4043 welding wire (Al-5Si wt.%) with a diameter of 1.2 mm. Table 1 shows the nominal chemical compositions of base metals and filler metal. V groove with a bevel angle of 45° was fabricated on the steel side and no groove on the aluminum side.

**Figure 1:**
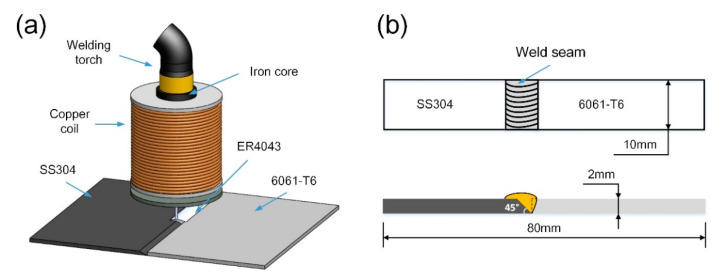
(**a**) Schematic diagram of the welding device; (**b**) detailed size of tensile test specimen.

The welding process was performed by an industrial robot (ABB, Zurich, Switzerland) and welding source (TPS 4000, Fronius, Pettenbach, Austria) in the normal CMT mode. Figure 1a shows the schematic diagram of the external magnetic field hybrid CMT welding process. The external magnetic field was generated through copper coils wound around a hollow iron core. The device was mounted on a welding torch coaxially. The direction of the magnetic field in the welding process was perpendicular to the welded workpiece. The magnetic intensity and alternating frequency can be controlled by the magnitude and frequency of coil current which can operate with control button on the magnetic control power source. Detailed experimental parameters are given in Table 2.

To analyze the quality of Al/steel joint by external magnetic field hybrid CMT welding process, the microstructure of the cross section was observed via scanning electron microscope (SEM, MERLIN Compact, Zeiss, Oberkochen, Germany) equipped with an energy-dispersive X-ray spectrometer (EDS). Transmission electron microscope (TEM, JEOL-2100, JEOL, Tokyo, Japan) was used to characterize the microstructure in detail. Finally, the specimens were cross-sectioned perpendicularly to the welding direction to investigate the mechanical properties of Al/steel butt joints, as shown in Figure 1b. The tensile tests were carried out by a 30 KN universal material testing machine (Instron 5967, Instron, Boston, MA, USA) at a loading rate of 0.5 mm/min. Three replicates were performed, and the average peak loads were reported to reflect the mechanical properties of the joints. The fractured surfaces of the specimens after tensile testing were examined by SEM with EDS and X-ray diffraction (XRD, DX-2700, Haoyuan, Dandong, China).

## 3. Results and Discussion

### 3.1. Weld Appearance

**Figure 2:**
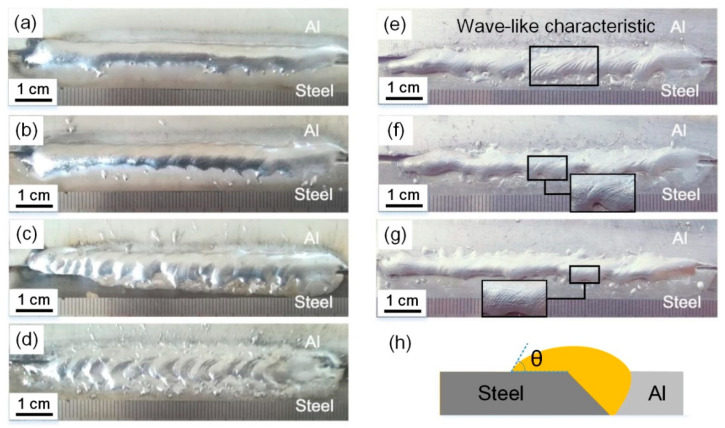
Weld appearances of Al/steel joints at different magnetic field parameters: (**a**) 0 A, 0 Hz; (**b**) 1 A, 0 Hz; (**c**) 2 A, 0 Hz; (**d**) 3 A, 0 Hz; (**e**) 2 A, 5 Hz; (**f**) 2 A, 10 Hz; (**g**) 2 A, 15 Hz; (**h**) schematic diagram of wetting angle (θ).

Figure 2 shows the weld appearances of the Al/steel butt joints obtained with different magnetic field parameters. Welding current of 73 A and welding speed of 6 mm/s with lower heat input were selected to reduce the formation of brittle Al/Fe IMCs. However, a narrow weld seam was obtained in the normal CMT welding process. The bonding area of aluminum weld metal on the steel surface was smaller. The spreadability improved and the weld width significantly increased with the introduction of the magnetic field. However, the larger welding spatters resulted in the loss of weld metal with excessive current of 3 A. The application of alternating frequency can stabilize the welding process and reduce welding spatters effectively, as shown in Figure 2e–g. Furthermore, a wave-like characteristic of weld surface was observed and the waves’ spacing was denser with the increase of alternating frequency.

**Figure 3:**
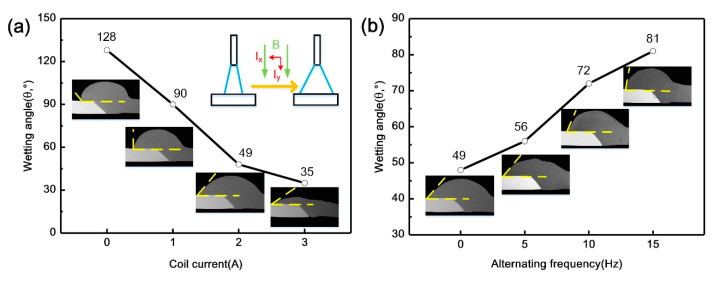
Wetting angle and cross sections of aluminum on steel surface at different magnetic field parameters: (**a**) Coil current; (**b**) alternating frequency.

In order to quantitatively describe the effect of coil current and alternating frequency on the weld appearance, the wetting angles were measured from the cross sections shown in Figure 3. In the normal CMT process in Figure 3a, the measured wetting angle is 128°, showing the poor wettability of weld metal on the steel surface. With the increase of coil current, the wetting angle tended to decrease with the same heat input. This was related to the arc heating redistribution on steel surface and the forced flowing of molten pool under the action of the external magnetic field. The arc plasma rotated due to the spiral motion of charged particles by the Lorentz force, which resulted in the increase of the arc width. Therefore, the heating area on the surface of the steel plate was expanded [25], which can help to break oxide film and promote wetting of weld metal. Additionally, diverging distribution of current lines existed in the molten pool from top to bottom as well, which was conducive to the spreading behavior of the molten pool driven by the electromagnetic force. Figure 3b shows the influence of alternating frequency on the wetting angle when the coil current was fixed at 2 A. The wetting angle increased with alternating frequency because of the weak flow of molten pool. The rotating direction of the arc and molten pool altered under the periodically alternating electromagnetic force. Therefore, the rotation radius of the arc and molten pool decreased due to the inertia force [28], which weakened the spreading dynamic of molten pool. The wave-like feature on the weld surface also indicated that the molten pool had a strong flow behavior before solidification.

### 3.2. Interfacial Microstructure

**Figure 4:**
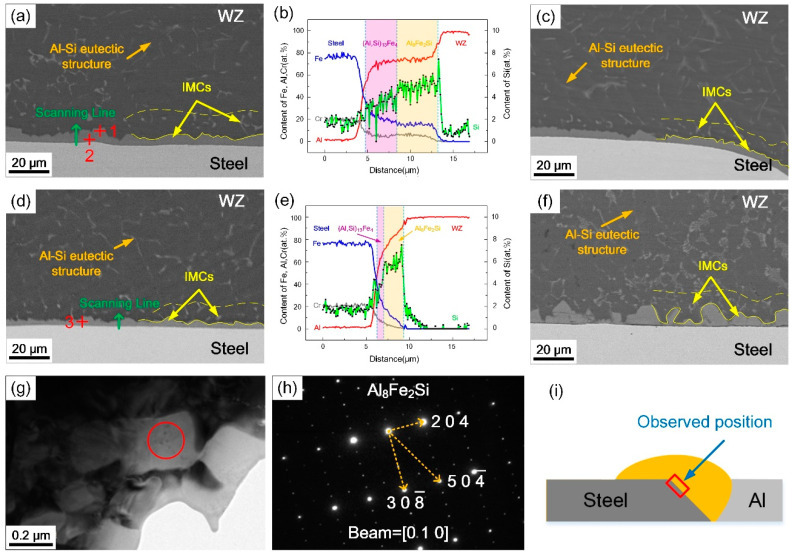
Microstructure, EDS line scanning results and selected area electron diffraction pattern of the WZ)/steel interface at different coil currents: (**a**,**b**) 0 A, 0 Hz; (**c**) 1 A, 0 Hz; (**d**,**e**) and (**g**,**h**) 2 A, 0 Hz; (**f**) 3 A, 0 Hz; (**i**) observed position.

Figure 4a–h shows the interfacial microstructures of Al/steel butt joints at different coil currents and the observed position is shown in Figure 4i. It can be seen that an approximate 6 μm thick IMC layer was formed at the Al/steel interface without the application of the magnetic field. The morphology of the IMC layer became smooth and the average thickness was gradually reduced to 3 μm with the increase of coil current, as shown in Figure 4c–d. This is because the peak temperature of the steel surface was decreased with the expansion effect of arc shape and molten pool, which weakened the capacity of element diffusion and suppressed the growth of the IMC layer. However, when the coil current further increased to 3 A, the thickness of IMC layer at the interface became uneven, as seen in Figure 4f. The excessively strong scouring action of rapid flow against steel surface and mass dissolution of Fe can be responsible for this phenomenon.

According to the EDS results listed in Table 3, the IMC layer obtained from the normal welding process was divided into two layers, including (Al, Si)_13_Fe_4_ phase adjacent to steel surface and Al_8_Fe_2_Si phase with less Fe content, which was consistent with the conventional results of Al/steel fusion welding process with Al-Si filler metal [10,11,12,13,14]. Under the coil current of 2 A, the IMC component was mainly Al_8_Fe_2_Si with more Si and less Fe. According to the EDS line scanning results in Figure 4b,e the thickness of IMC layer reduced and the elemental concentration gradient cross interface was steeper due to the presence of the magnetic field. The (Al, Si)_13_Fe_4_ phase became poor and Si content increased in the IMC layer. In order to determine the phase of the IMC layer more accurately, TEM micrographs with corresponding SADP taken from the welding zone (WZ)/steel interface are shown in Figure 4g–h. The results show that the IMC layer was identified as Al_8_Fe_2_Si phase with hexagonal structure. 

**Figure 5:**
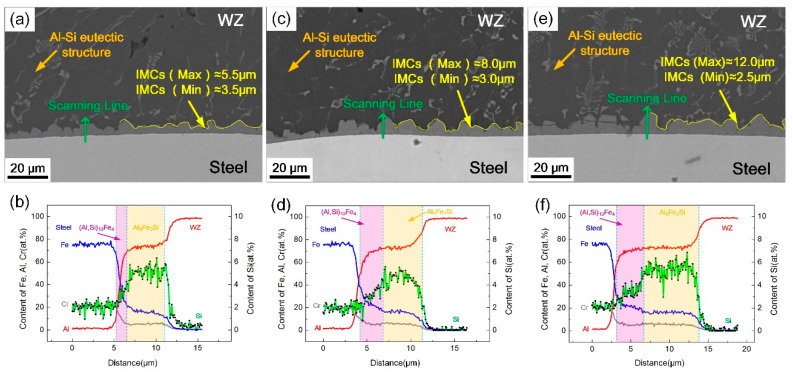
Microstructure and EDS line scanning results of WZ/steel interface at different alternating frequencies: (**a**,**b**) 2 A, 5 Hz; (**c**,**d**) 2 A, 10 Hz; (**e**,**f**) 2 A, 15 Hz.

In addition, changing the alternating frequency of the magnetic field will also affect the interfacial morphology. Figure 5 shows the interfacial microstructures of Al/steel joints in different alternating frequencies, when the coil current was fixed at 2 A. It was observed that the IMC layer became thicker and uneven with the increase of alternating frequency. Under the action of alternative electromagnetic force and rotating arc, the magnetic frequency intensified the oscillating behavior of molten pool at the front interface, which caused the nonuniform accumulation of local elements and formed a serrated compound layer during rapid cooling. Meanwhile, the rotation radius of weld arc decreased with the alternating magnetic field due to inertia force, which increased the heat input per unit area on the Al/steel interface, resulting in an increase in the IMC layer thickness [26]. Therefore, the larger the alternating frequency was, the greater the average thickness of the IMC layer became. The corresponding line scanning results show that the thickness of (Al, Si)_13_Fe_4_ layer with lower Si content increased. However, its proportion was still lower than the normal welding process, indicating that the magnetic field can inhibit the growth of (Al, Si)_13_Fe_4_ layer and promote Si aggregating at the interface.

**Figure 6:**
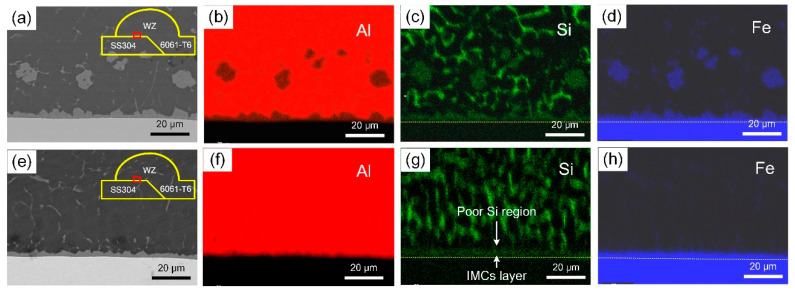
EDS element distribution maps and distribution of Al, Si and Fe in different magnetic field parameters: (**a**–**d**) 0 A, 0 Hz; (**e**–**h**) 2 A, 0 Hz.

SEM amplifying images of the IMCs and the distribution of Al, Si and Fe near the interface are presented in Figure 6. For a normal CMT welding process, Si concentrated along the reticulate grain boundary of Al-Si eutectic structure and aggregated at the Al/steel interface where distributed a lower chemical potential. A long-distance diffusion of Fe atoms can be observed at the interface, as well as block-shaped distribution in the welding zone, which indicated that Fe had a great capacity for diffusion to aluminum weld.

The thickness of the IMC layer decreased to 3 μm and became uniform with the magnetic field. The diffusion of Si was directional toward the steel surface from aluminum weld. Meanwhile, a visible poor Si region occurred near the IMC layer, which can be an evidence of the enrichment effect of Si under the action of magnetic field as well. This can be attributed to the continuous transportation of Si atoms to the interface with the rapid flow of molten pool. Conversely, the diffusion degree of Fe was reduced mainly caused by the lower peak temperature. The silicon-rich IMC layer acted as an interdiffusion barrier of Fe and Al atoms as well [14].

**Figure 7:**
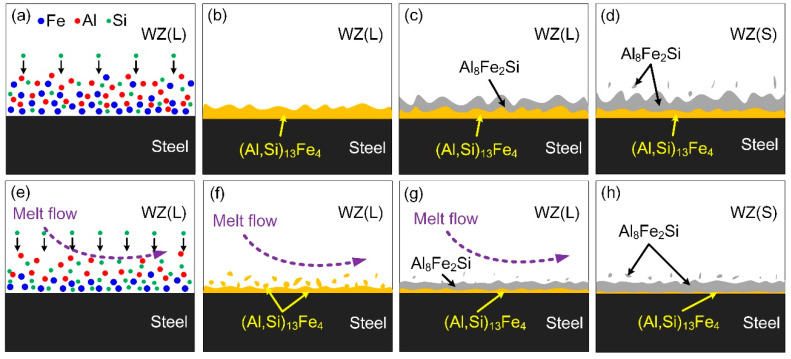
Schematic diagram of IMC layer formation: (**a**–**d**) In the normal welding process; (**e**–**h**) with axial magnetic field assisted.

According to the interfacial reactions [29], the IMC layer formations in the normal CMT process and magnetic field assisted welding were contrastively analyzed to clarify the action mechanism of magnetic field on the interfacial reaction, as presented in Figure 7. In the normal CMT process, the steel dissolved in the molten pool while Si atoms diffused rapidly and aggregated at the interface. When the temperature fell to 1020 °C, (Al, Si)_13_Fe_4_ phase was primarily produced at the interface with Si atoms substitutional solubilized. Al_8_Fe_2_Si phase was formed adjoining to the (Al, Si)_13_Fe_4_ layer with the reaction of liquid aluminum and (Al, Si)_13_Fe_4_ phase, when the temperature of the interface cooled to 620 °C. The Al_8_Fe_2_Si layer grew to WZ, while the (Al, Si)_13_Fe_4_ layer was consumed to a thinner thickness. Finally, Fe and Si atoms far in the weld formed block-shaped or rod-shaped Al_8_Fe_2_Si phase with the solidification of aluminum.

With the application of the external axial magnetic field, the diffusion distance of Fe atoms was shortened and more Si atoms aggregated at the interface. (Al, Si)_13_Fe_4_ layer was generated and grew in the temperature range of 1020–620 °C. However, the production of (Al, Si)_13_Fe_4_ declined on account of the shortening of the residence time at this temperature range. Furthermore, (Al, Si)_13_Fe_4_ layer was constantly smashed by the strong scouring action of rapidly flowing molten pool. The smashed compounds and remaining (Al, Si)_13_Fe_4_ layer reacted with liquid aluminum to form Al_8_Fe_2_Si phase, when the interfacial temperature decreased to 620 °C. The Al_8_Fe_2_Si layer grew with the consumption of liquid aluminum and remaining (Al, Si)_13_Fe_4_ phase. Hence rare (Al, Si)_13_Fe_4_ remained and the main component of the IMC layer was Al_8_Fe_2_Si phase, which had an improvement on mechanical properties.

### 3.3. Tensile Strength and Failure Behavior

**Figure 8:**
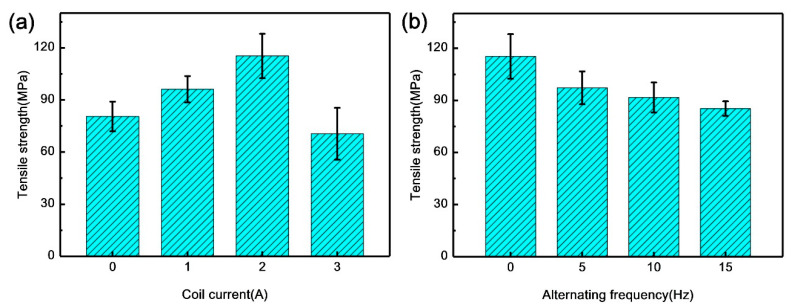
Tensile strength of the specimens with different various field parameters: (**a**) Coil current; (**b**) alternating frequency.

Figure 8 shows the tensile strength of the specimens with various magnetic field parameters, which was reported to represent the mechanical properties of the joints. The results show that the tensile strength increased with the rise of coil current, although it dropped when the coil current increased to 3 A. The variation of the tensile strength was positively associated with IMC layer thickness with the increase of coil current. Additionally, the increase of the bonding area can further improve the tensile strength. As shown in Figure 8b, the tensile strength decreased with the rise of alternating frequency. The phenomenon can be attributed to the increase of the thickness of the IMC layer and (Al, Si)_13_Fe_4_ phase content formed at higher temperature.

**Figure 9:**
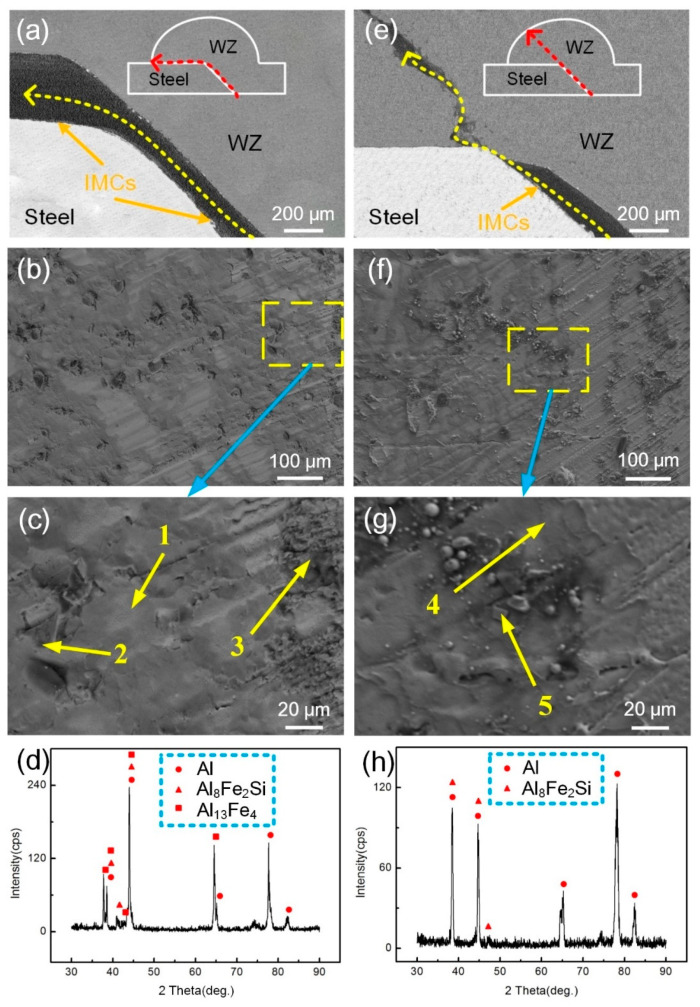
Fracture path, fractography and XRD analysis results of steel side of tensile specimens with various welding conditions: (**a**–**d**) 0 A, 0 Hz; (**e**–**h**) 2 A, 0 Hz.

Figure 9 shows the fracture path and fractography of the welded joints with various welding conditions. Chemical compositions of location 1–5 obtained by EDS detections are listed in Table 4. In Figure 9a–c, the joints fractured along the (Al, Si)_13_Fe_4_ layer without the magnetic field. The joint was characterized by brittle fracture with flat fracture surface and no dimples. The fracture surface was smooth and (Al, Si)_13_Fe_4_ compound was identified. The fracture morphology in region 2 was α-Al phase with a content of 98.1 at.% Al and 1.9 at.% Si. In region 3, rough fracture surface was located on the bottom of the groove where the crack source was. The component was made up of 72.6 at.% Al, 8.3 at.% Si and 19.1 at.% Fe, implying that this IMC was Al_8_Fe_2_Si phase. To further confirm the phase, XRD analysis was performed and the results are shown in Figure 9d. The results show that Al, Al_8_Fe_2_Si and Al_13_Fe_4_ were found on the fracture surface.

In Figure 9e–g, a mixed fracture path including Al_8_Fe_2_Si layer and WZ was obtained with the introduction of the magnetic field. The typical morphology of the fracture surface was composed of Al_8_Fe_2_Si phase and α-Al flakes that distributed on the Al_8_Fe_2_Si IMC layer. The results indicate that the change of the IMC components from (Al, Si)_13_Fe_4_ to Al_8_Fe_2_Si phase, effectively improving the tensile strength of the joints. The XRD analysis in Figure 9h further verified the existence of these two products.

## 4. Conclusions

In this study, an axial magnetic field was introduced to the CMT butt-welding of 6061 aluminum alloy and 304 stainless steel with ER4043 filler metal. The effects of the external axial magnetic field on weld appearance, interfacial microstructure, and tensile strength of the resultant joints were investigated. The conclusions are summarized as follows:

(1) With the increase of coil current, the spreadability of the molten filler was improved and the wetting angle decreased from to 128° to 35°. The wetting facilitation effect on the molten pool was reduced with the increase of alternating frequency. The wave-like characteristic occurred on the weld surface after the solidification of the fiercely stirred molten pool.
(2) With the assistance of the magnetic field, the growth of the IMC layer was inhibited and the transformation of the IMC layer from (Al, Si)13Fe4 to Al8Fe2Si phase was promoted, which was beneficial to the mechanical properties of Al/steel joints. However, excessive coil current and the increase of alternating frequency resulted in a thicker and uneven IMC layer.
(3) The maximum tensile strength reached 130.2 MPa with the coil current and alternating frequency of 2 A and 0 Hz, increasing by a 61.6% contrast with the normal CMT welding process. The fracture path changed from the (Al, Si)13Fe4 layer to Al8Fe2Si layer-welding zone.
(4) In future work, the effect of the magnetic field on the dynamic behavior of the molten pool will be investigated by numerical analysis combined with experimental observation.

## Figures and Tables

**Table 1:** Nominal chemical compositions of base metal and filler metal.

Materials	Al	Fe	Si	Cr	Mn	Ni	Mg	Cu	Ti	C
6061-T6	Bal.	0.7	0.4–0.8	-	0.15	-	08–1.2	0.15–0.4	-	1.0
SS304	-	Bal.	1.0	18-20	2.0	8.0–11	-	-	0.08	0.08
ER4043	Bal.	0.8	4.5–6.0	-	0.3	-	0.05	0.3	0.2	-

**Table 2:** Detailed experimental parameters.

Wire Feeding Speed (M/Min)	Welding Current (A)	Welding Speed (mm/s)	Coil Current (A)	Magnetic Intensity (mT)	Alternating Frequency (Hz)
4.1	73	6	0	0	0
4.1	73	6	1	6.4	0
4.1	73	6	2	13.3	0
4.1	73	6	3	19.5	0
4.1	73	6	2	13.3	5
4.1	73	6	2	13.3	10
4.1	73	6	2	13.3	15

**Table 3:** EDS spot scanning results of zones 1–3 in Figure 4.

Location	Al (at.%)	Si (at.%)	Fe (at.%)	Possible Phase
1	75.5	6.1	18.4	Al_8_Fe_2_Si
2	69.8	5.9	24.3	(Al, Si)_13_Fe_4_
3	72.6	8.3	19.1	Al_8_Fe_2_Si

**Table 4:** EDS spot scanning results of zones 1–5 in Figure 9.

Location	Al (at.%)	Si (at.%)	Fe (at.%)	Possible Phase
1	69.7	5.8	24.5	(Al, Si)_13_Fe_4_
2	98.1	1.9	-	α-Al solid
3	72.6	8.3	19.1	Al_8_Fe_2_Si
4	74.2	8.0	17.8	Al_8_Fe_2_Si
5	92.8	7.2	-	α-Al solid
